# Match the Metatarsal Heads: A Case of All-Metatarsal Fractures After a Motorcycle Accident

**DOI:** 10.7759/cureus.39659

**Published:** 2023-05-29

**Authors:** Nuri Ayoglu, Muhammed Yusuf Afacan, Mahmut Kursat Ozsahin, Ali Seker

**Affiliations:** 1 Department of Orthopaedics and Traumatology, Istanbul University-Cerrahpasa, Cerrahpasa Medical Faculty, Istanbul, TUR

**Keywords:** motorcycle accident, distal metatarsal fracture, first metatarsal proximal fracture, satisfactory recovery potential, importance of surgery, k-wire fixation, closed reduction, open reduction, adolescence polytraumatic bone fractures, metatarsal fractures

## Abstract

Metatarsal bone fractures are one of the most frequent foot injuries, especially after motor vehicle accidents in children. This case report briefly demonstrated a rare instance of pediatric all-metatarsal fractures of the left foot in an adolescent patient with polytrauma after a motorcycle accident. This case report illustrated the surgical procedure's potential for healing pediatric foot fractures in teenage patients after polytrauma. In the examination of a 16-year-old male patient brought to the emergency department after a motorcycle accident, we detected a right foot third finger proximal phalanx open fracture, right foot fourth finger proximal phalanx fracture, left foot first metatarsal proximal fracture, left foot second, third, fourth, and fifth metatarsal distal fractures, left foot cuboid, and navicular bone fractures. The patient's left foot's metatarsals were all fractured. The posterolateral wall fracture of the patient's right maxilla was also detected. All metatarsals were displaced, the second metatarsal paired with the third, etc., and because of this displacement, the closed reduction was impossible, and even the open reduction was challenging to reach the correct pairs. We performed closed reduction and fixation with Kirschner wire for the left foot's first metatarsal fracture and open reduction and fixation with Kirschner wire for the left foot's second, third, and fourth metatarsal distal fractures. We also performed closed reduction and fixation with Kirschner wire for the right foot third and fourth proximal phalanx fractures. We observed callus formation in the sixth week and removed the patient's K-wires. At eight weeks, the X-ray demonstrated the correct alignment of all metatarsals. The proper alignment of all metatarsals and the full range of motion of all foot and ankle joints were achieved with early surgical intervention, open reduction, and timely rehabilitation. This case also emphasizes the importance of open reduction in such irreducible and heavily displaced cases of multiple fractures as all-metatarsal fractures and contributes to the literature with a specific treatment modality in the case of all-metatarsal fractures lacking in the literature.

## Introduction

Foot bone fractures constitute 5-8% of pediatric fractures [1]. One of the most frequent injuries to the foot is metatarsal bone fractures. The metatarsal bones account for 61% of all foot fractures in the pediatric age group. Most pediatric metatarsal fractures occur at the fifth (41%) and first (19%) rays due to their anatomical exposure [2]. Although good results are generally the results of conservative treatment in pediatric foot fractures, some of these fractures may have poor results despite anatomical reduction and internal fixation [3]. Determining which fractures need surgical treatment, avoiding compartment syndrome, early detection of complications like post-traumatic foot deformities and avascular necrosis, and controlling their treatment are the biggest challenges in managing pediatric foot fractures [4]. Even though each metatarsal injury pattern is distinct, and the fractures should be treated according to the surgeon's clinical opinion, there seem to be absolute and relative indications for surgical intervention. The relative criteria for performing surgery in metatarsal shaft fractures seem to be young age and multiple fractures. However, open and articular fractures may be absolute indications for surgery. Moreover, even with successful surgery, the surgeon should follow up with the patient frequently with radiographs. The main reason is 15.4% of the patients may have delayed bony union [5]. With this case report, we aimed to demonstrate a rare instance of heavily displaced pediatric all-metatarsal fractures of the left foot in a teenage patient with polytrauma after a motorcycle accident. This case report also illustrated the surgical procedure's potential for healing pediatric all-metatarsal fractures in teenage patients after polytrauma. The failure of closed reduction and the success of open reduction in heavily displaced and irreducible pediatric all-metatarsal fractures to maintain the proper alignment was also depicted in this case report. We also attempted to review and cite some of the existing studies despite being too few on multiple or all-metatarsal fractures, which could help position this case within the current literature.

## Case presentation

**Figure 1:**
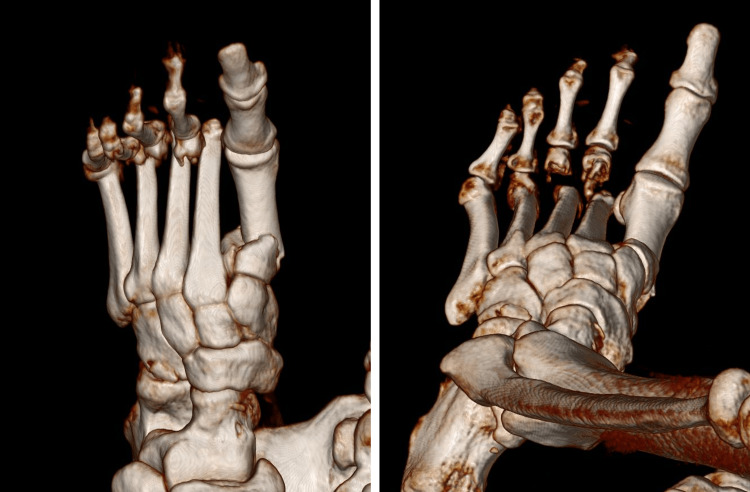
Three-dimensional reconstruction of the left foot's computed tomography Three-dimensional reconstruction of the left foot's computed tomography showing left foot first metatarsal proximal fracture, left foot second, third, fourth, and fifth metatarsal distal displaced fractures, left foot cuboid, and navicular bone fractures. The patient's left foot's metatarsals were all fractured.

**Figure 2:**
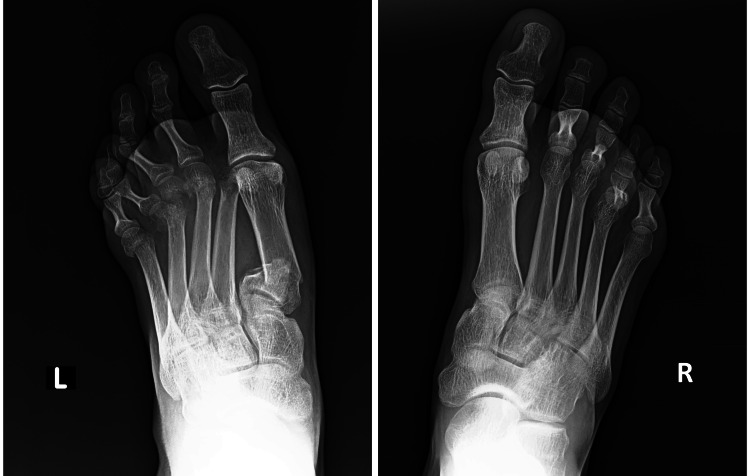
Anteroposterior X-ray view of the left and right foot after the accident Anteroposterior X-ray view of the left and right foot after the accident showing right foot third finger proximal phalanx open fracture, right foot fourth finger proximal phalanx fracture, left foot first metatarsal proximal fracture, left foot second, third, fourth, and fifth metatarsal displaced fractures, left foot cuboid, and navicular bone fractures. The patient's left foot's metatarsals were all fractured.

A 16-year-old male patient without past medical history was brought to the emergency department after a motor vehicle accident. He was a non-smoker and he didn't use any drugs. In the physical examination, there was widespread pain, swelling, ecchymosis, and deformity in the dorsum of the left foot and right third and fourth finger and also in the right part of the face. The visual analogue scale score was 7 for the right part of the face and 7 for the right foot and 10 for the left foot. The left foot movements were painful, limited, and not well-determined due to the pain. There was no active bleeding. There was a 1 cm skin lesion compatible with an open fracture on the dorsal side of the proximal phalanx of the third toe of the right foot. There was no neurovascular deficit in both lower extremities, and the tendons were intact. The patient can slightly dorsiflex the left foot's ankle and both toes despite the pain. After the X-ray imaging and computed tomography, we detected the right foot third finger proximal phalanx open fracture, the right foot fourth finger proximal phalanx fracture, the left foot first metatarsal proximal fracture, the left foot second, third, fourth, and fifth metatarsal distal fractures, the left foot cuboid, and navicular bone fractures. The patient's left foot's metatarsals were all fractured and heavily displaced (Figures 1-2).

**Figure 3:**
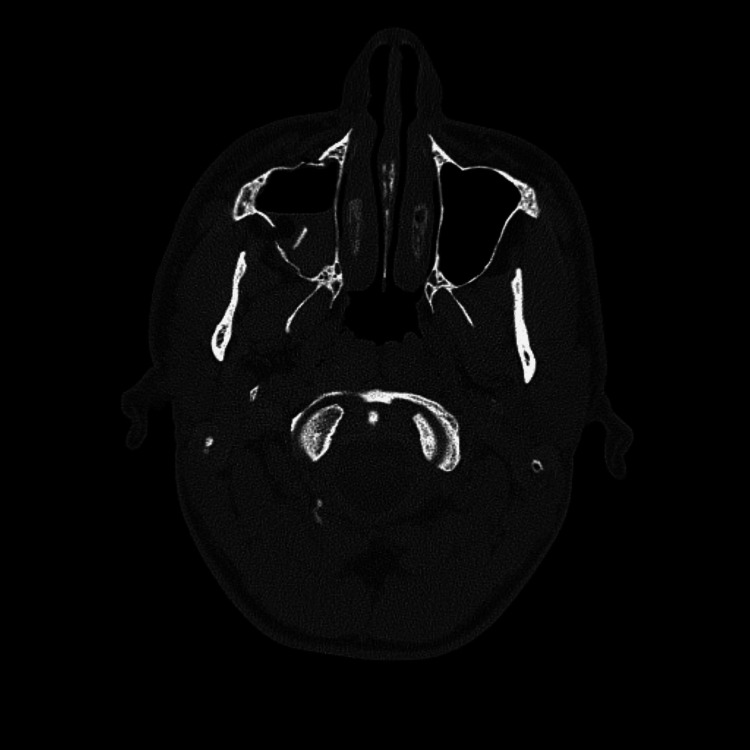
Cranial computed tomography of the patient after the trauma Transverse section of the cranial computed tomography of the patient after the trauma indicated a displaced fracture at the right posterolateral wall of the maxilla and a resulting hematoma in the maxillary sinus.

In addition, the patient had right periorbital ecchymosis and a fracture in the right posterolateral wall of the maxilla (Figure 3).

**Figure 4:**
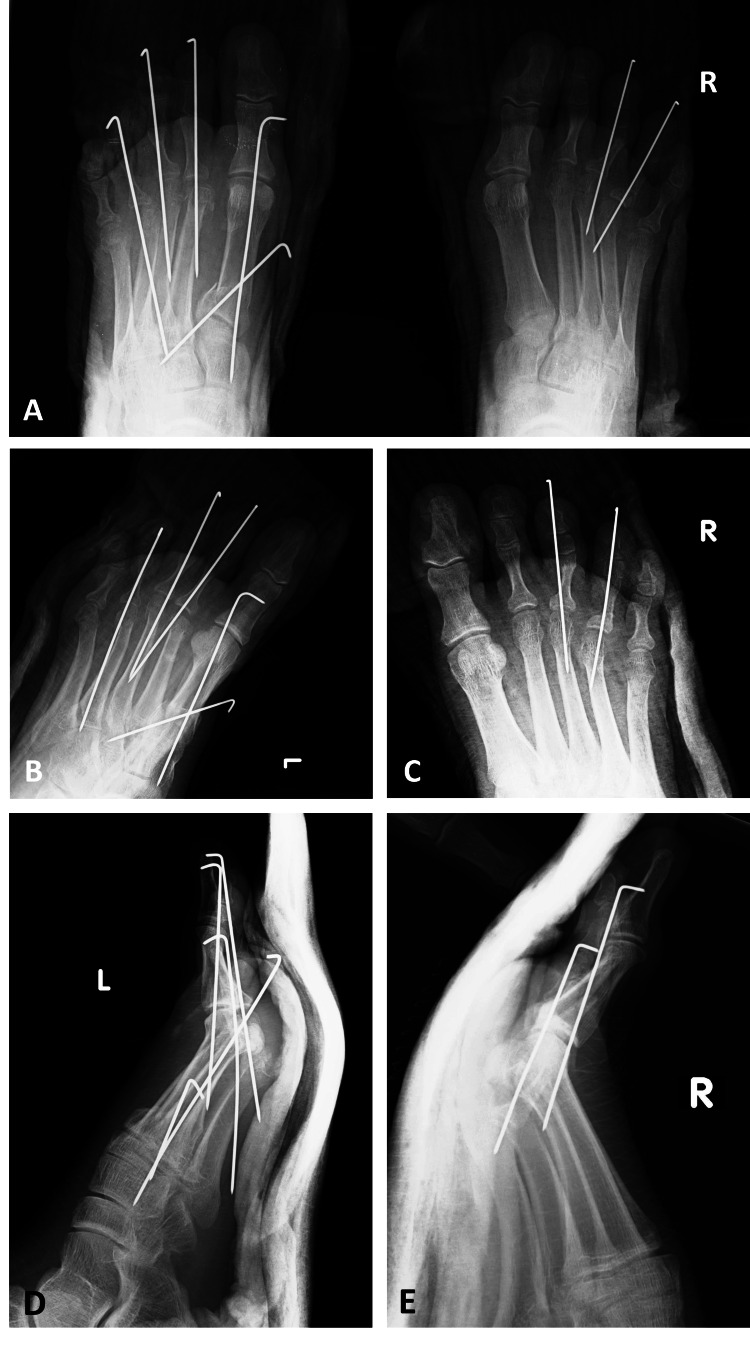
The left and right foot's X-ray views directly after the surgery A: The left and right foot's anteroposterior X-ray view directly after the surgery showed the open and closed reduction with K-wire fixation. B: The left foot's oblique X-ray view directly after the surgery showed the open and closed reduction with K-wire fixation. C: The right foot's oblique X-ray view directly after the surgery showed the closed reduction with K-wire fixation. D: The left foot's lateral X-ray view directly after the surgery showed the open and closed reduction with K-wire fixation. E: The right foot's lateral X-ray view directly after the surgery showed the closed reduction with K-wire fixation.

Cefazolin 3x1000 mg, gentamicin 2x80 mg, and metronidazole 3x500 mg treatment were applied via the IV route immediately to the patient, and tetanus prophylaxis was administered. As pain-killer paracetamol 3x1000 mg, dexketoprofen 2x50 mg, and tramadol 3x50 mg were applied via the IV route in case of need. Two short leg splints were applied bilaterally, and we transferred the patient to the orthopedics and traumatology inpatient clinic. As soon as the fasting period recommended by the anesthesiologists was over, we took the patient into the operating theatre. We performed closed reduction and fixation with Kirschner wire for the left foot's first metatarsal fracture and open reduction and fixation with Kirschner wire for the second, third, and fourth metatarsal distal fractures of the left foot. We also performed closed reduction and fixation with Kirschner wire for the right foot third and fourth proximal phalanx fractures (Figure 4).

**Figure 5:**
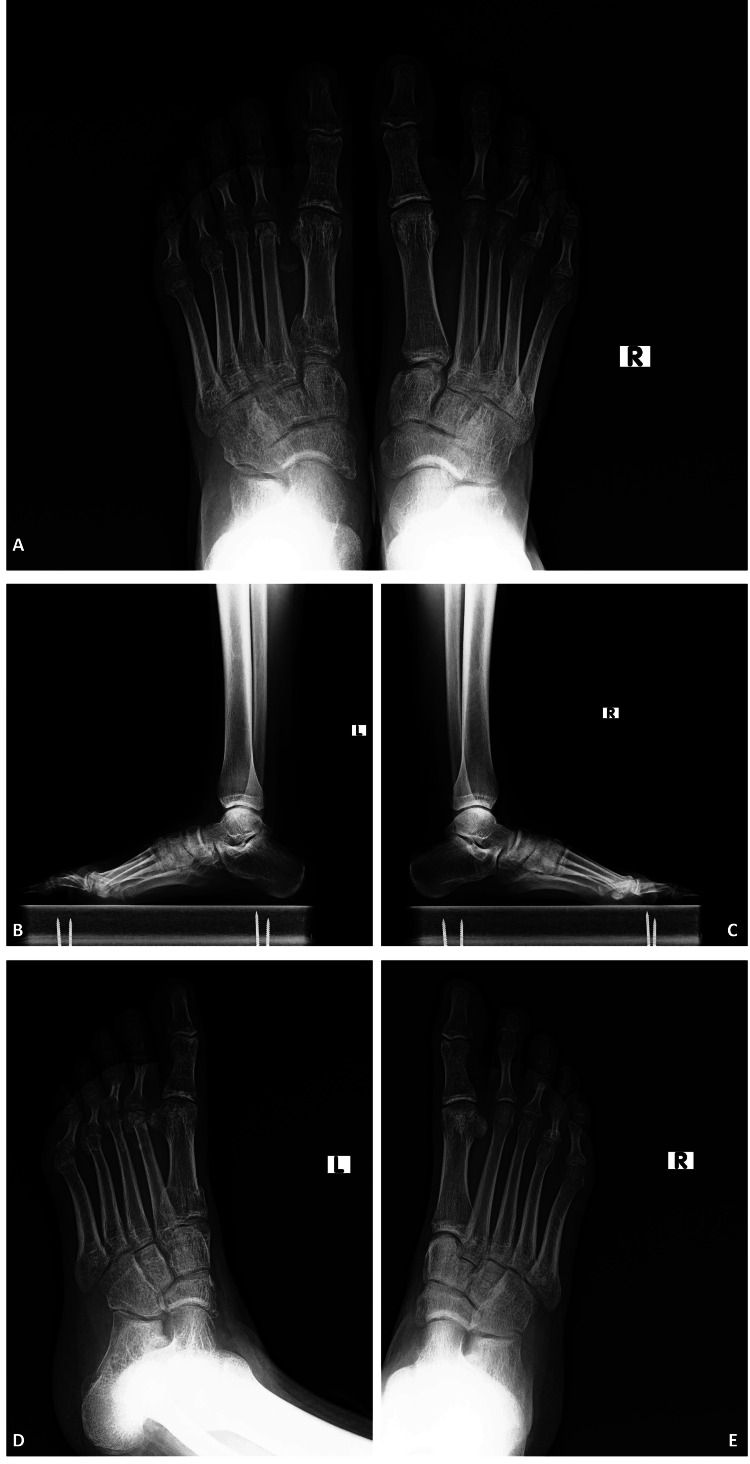
The left and right foot's X-ray images at eight weeks postoperatively A: The left and right foot's anteroposterior X-ray image at eight weeks postoperatively showed the proper alignment of all metatarsals and phalanges. B: The left foot's lateral X-ray image at eight weeks postoperatively showed the proper alignment of all metatarsals and phalanges. C: The right foot's lateral X-ray image at eight weeks postoperatively showed the proper alignment of all metatarsals and phalanges. D: The left foot's oblique X-ray image at eight weeks postoperatively showed the proper alignment of all metatarsals and phalanges. E: The right foot's oblique X-ray image at eight weeks postoperatively showed the proper alignment of all metatarsals and phalanges.

**Figure 6:**
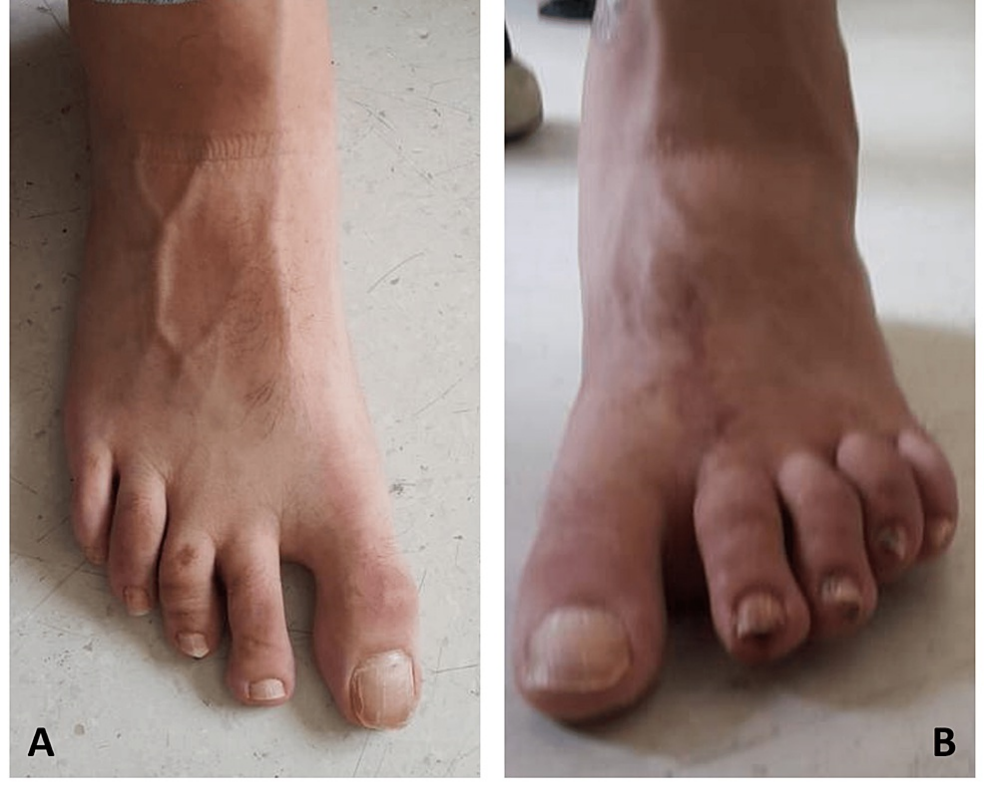
The left and right foot's clinical images at eight weeks postoperatively A: The right foot's clinical images at eight weeks postoperatively showed the proper alignment of all metatarsals and phalanges. B: The left foot's clinical images at eight weeks postoperatively showed the proper alignment of all metatarsals and phalanges.

**Figure 7:**
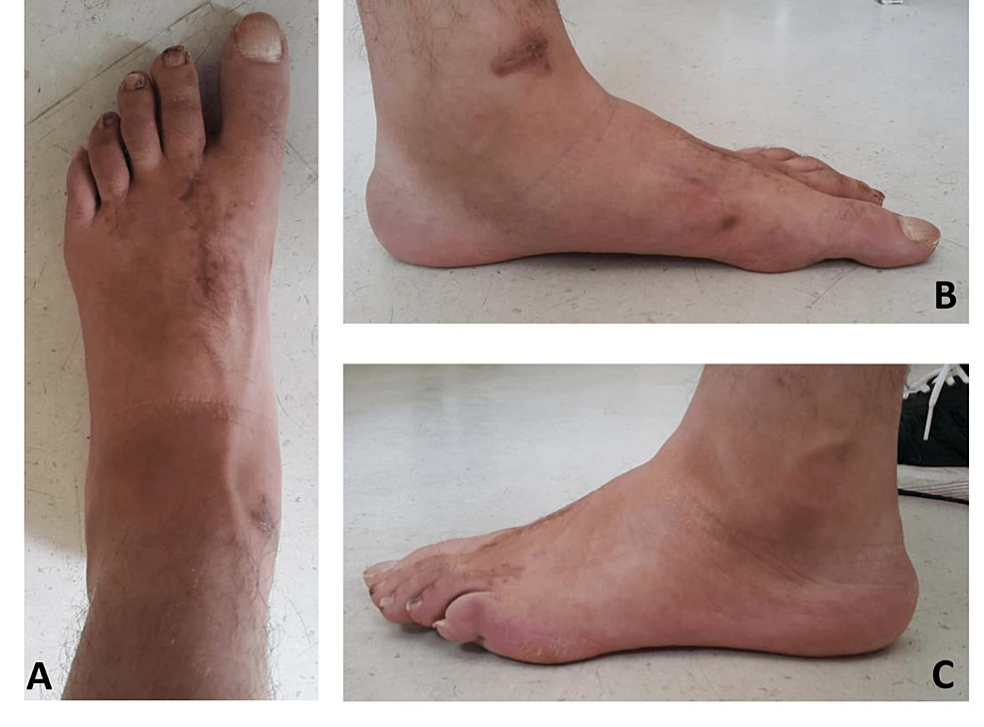
The left foot's clinical images at eight weeks postoperatively showed the proper alignment of all metatarsals and phalanges

There were no pre- or postoperative complications and no neurovascular deficits. The patient got discharged on the third postoperative day with a short leg splint. We strongly warned the patient not to step on his foot and not to give full weight till the end of the sixth week. We removed the patient's stitches the second week after the operation, and there were no wound problems. We observed callus formation in the sixth week and removed the patient's K-wires. The proper and correct alignment of all metatarsals was shown both clinically and radiologically in the X-ray at eight weeks (Figures 5-7).

We observed no infection or wound healing problem in the patient's follow-up. There were no patient complaints on the right and left foot in the follow-up at eight weeks. However, there was an extension limitation in the left toe in the examination. The patient was then referred to the physical therapy and rehabilitation department at the end of the eighth week to restore range of motion and muscle strength and prevent atrophy for four weeks. With the help of the full range of motion and isometric exercises above and below the level of injury, the full range of motion of all metatarsophalangeal, proximal interphalangeal, and distal interphalangeal joints of both feet was obtained, and the atrophy of the muscles was reversed. The proper and correct alignment of all metatarsals and a full range of motion of all foot and ankle joints were achieved at the end of the third month with early surgical intervention and timely rehabilitation with the help of the open reduction procedure after the closed reduction procedure had failed. The patient fully returned to sports at the end of the sixth month and maintained his life as if he hadn't encountered any all-metatarsal fracture or trauma emphasizing the success of early surgery and open reduction for the correct bony match.

## Discussion

Serious injuries, particularly to the foot, are still frequently caused by motorcycle accidents. A metatarsal fracture is the most frequent foot injury, but we should also suspect a concomitant ailment after those accidents as in our case [6]. In our case, the patient had all-metatarsal fractures of the left foot, an open fracture of the right foot's phalanges, and the patient also had a fracture in the right posterolateral wall of the maxilla treated conservatively.

The mortality rate in such foot fractures is low. Despite the low fatality incidence of these foot injuries, we should evaluate and treat them immediately to prevent permanent disabilities [6]. In our case, the patient was operated on immediately after the motorcycle accident. Thus, we achieved the proper alignment of all metatarsals surgically. Open reduction and K-wire fixation eased the bony union and prevented the patient from nonunion and malunion. The closed reduction failed in maintaining the accurate match of the metatarsal raws. Therefore, an open reduction was compulsory to match the proximal and distal bony ends of the metatarsals.

Children should be followed up frequently radiographically for the bony union after displaced fractures. The main reason is 15.4% of the patients may have delayed bony union [5]. In the follow-up, we observed a normal bony union in the second month. In the radiographs taken in the third, sixth, and 12th months, we detected neither a nonunion nor a malunion. The main reason for such recovery is the early surgical intervention with timely rehabilitation. Despite the presence of multiple foot fractures in our patient and the fact that the second, third, fourth, and fifth metatarsal distal fractures coming into contact with the ground during walking may cause chronic pain and deformity in the foot, especially during walking in the future, surgical treatment with the open reduction and K-wire fixation in the early period seems to be satisfactory in maintaining the correct match of all metatarsals.

As a result of our literature review, the case of fracture of all metatarsals was very rare, and cases with two or a maximum of three metatarsal fractures were available in the literature. In addition, treatment modalities for each metatarsal fracture were generally mentioned separately in the literature [7-9]. Our study is unique both because it is a case with a fracture of all metatarsals and because it answers the questions of how surgical treatment should be in a case with a fracture of all metatarsals and how to correctly match the metatarsals surgically. Moreover, our study will contribute to the literature both in terms of surgical technique and the prognosis of the patient in the case of all-metatarsal fractures lacking in the literature.

## Conclusions

This case report briefly demonstrated a rare instance of pediatric all-metatarsal heavily displaced fractures of the left foot in an adolescent polytraumatic patient after a motorcycle accident causing a mismatch of the metatarsals. The proper alignment of all metatarsals and a full range of motion of foot and ankle joints were achieved with early surgical intervention due to the open reduction and K-wire fixation of the metatarsals followed by timely rehabilitation. Mismatch of the left foot's metatarsals after an all-metatarsals fracture caused a failure in closed reduction, which made the open reduction a necessity to obtain the proper alignment and correct match of the distal and proximal bony ends. Even with the open reduction, it was a great constraint to bring the bone ends together due to the all-displacement of the left foot's all metatarsals. However, the patient maintains his life as if he didn't encounter any trauma which emphasizes the success of early surgery and the importance of open reduction for the correct bony match. This case is of great importance because the simultaneous all-metatarsal fracture and its specific treatment and prognosis lack in the literature.
